# Effects of sports drinks on the maintenance of physical performance during 3 tennis matches: a randomized controlled study

**DOI:** 10.1186/s12970-014-0046-7

**Published:** 2014-09-02

**Authors:** Thibault Brink-Elfegoun, Sébastien Ratel, Pierre-Marie Leprêtre, Lore Metz, Gael Ennequin, Eric Doré, Vincent Martin, David Bishop, Nicolas Aubineau, Jean-François Lescuyer, Martine Duclos, Pascal Sirvent, Sébastien L Peltier

**Affiliations:** 1 Division of Sport Medicine and Biology of Physical Activity, University of Athens Faculty of Physical Education and Sport Science, Antistasis 41, Athens, 17237, Dafni, Greece; 2 Clermont Université, Université Blaise Pascal, EA 3533, Laboratoire des Adaptations Métaboliques à l’Exercice en conditions Physiologiques et Pathologiques (AME2P), Bâtiment Biologie B, 24 avenue des Landais, Aubière Cedex, F-63171, France; 3 Laboratoire de Recherche Adaptations Physiologiques à l’Exercice et Réadaptation à l’Effort, EA-3300, Faculté des Sciences du Sport, Université de Picardie Jules Verne, Amiens, France; 4 Institute of Sport, Exercise and Active Living (ISEAL), Victoria University, Melbourne, Australia; 5 College of Sport and Exercise Science, Victoria University, Melbourne, Australia; 6 Department of Research, Laboratoire Lescuyer, Nutratletic, Aytré, France; 7 Department of Sport Medicine and Functional Explorations, University-Hospital (CHU), G. Montpied Hospital, Clermont-Ferrand F-63003, France; 8 INRA, UMR 1019, Clermont-Ferrand F-63001, France; 9 University Clermont 1, UFR Médecine, Clermont-Ferrand F-63001, France; 10 CRNH-Auvergne, Clermont-Ferrand F-63001, France

**Keywords:** Tennis, Nutrition, Fatigue, Performance, Sports drinks

## Abstract

**Background:**

Tennis tournaments often involve playing several consecutive matches interspersed with short periods of recovery.

**Objective:**

The objective of this study was firstly to assess the impact of several successive tennis matches on the physical performance of competitive players and secondly to evaluate the potential of sports drinks to minimize the fatigue induced by repeated matches.

**Methods:**

This was a crossover, randomized controlled study. Eight male regionally-ranked tennis players participated in this study. Players underwent a series of physical tests to assess their strength, speed, power and endurance following the completion of three tennis matches each of two hours duration played over three consecutive half-days (1.5 day period for each condition). In the first condition the players consumed a sports drink before, during and after each match; in the second, they drank an identical volume of placebo water. The results obtained were compared with the third ‘rest’ condition in which the subjects did not play any tennis. Main outcomes measured were maximal isometric strength and fatigability of knee and elbow extensors, 20-m sprint speed, jumping height, specific repeated sprint ability test and hand grip strength.

**Results:**

The physical test results for the lower limbs showed no significant differences between the three conditions. Conversely, on the upper limbs the EMG data showed greater fatigue of the *triceps brachii* in the placebo condition compared to the rest condition, while the ingestion of sports drinks attenuated this fatigue.

**Conclusions:**

This study has demonstrated for the first time that, when tennis players are adequately hydrated and ingest balanced meals between matches, then no large drop in physical performance is observed even during consecutive competitive matches.

**Trial registration:**

ClinicalTrials.gov: NCT01353872.

## Introduction

Tennis tournaments are quite complex due to their variability in terms of exercise duration and the type of effort required. One feature of competitive tennis is that the season is relatively long and that the ranking system pushes players to compete all year long. During a competition, players must sometimes play one or two matches a day on consecutive days. For many reasons the duration and intensity of these matches are highly variable, but it is not uncommon to see matches continue beyond three hours [1],[2] and various studies have shown a drop in high-level tennis performance during extended matches [3]–[6]. Under these conditions, optimum recovery methods are needed to maintain a high level of performance over the duration of a match, tournament or season. Among the strategies used, nutrition appears to be an important element to consider [7].

The majority of studies on the impact of nutritional strategies on tennis performance have been conducted by taking measurements during or at the end of long matches. Some studies have suggested a beneficial effect of carbohydrates during prolonged tennis matches [4],[5],[8]–[10]. Caffeine has also been suggested as positively affecting performance, although the number of relevant studies is very limited [4],[5],[9]. Among less common nutritional strategies, one study has also demonstrated a beneficial effect of sodium bicarbonate [6]. On the other hand, creatine supplementation did not appear to lead to positive effects on tennis performance [11],[12].

To our knowledge, no study has evaluated the effects of nutritional strategies on physical performance in the days following a series of matches, despite this being the reality of competitive tennis. Furthermore, studies conducted in the field of tennis nutrition have only been interested in the isolated effects of nutritional strategies before or during the match. However, it is increasingly common for competitive athletes to use sports drinks before, during and after matches to help maintain their performance over the duration of a tournament [13]. Different types of commercial beverages, specifically formulated to meet the needs of athletes before, during or after exercise, have been developed and introduced into the market in recent years, even though the claimed benefits of these advertised products have usually not been scientifically proven [14].

Recently, we have conducted a controlled, randomized, double-blind study to evaluate the impact of ingesting specially formulated pre-exercise, endurance, and recovery sports drinks on glycaemia and tennis performance indices during a simulated tennis tournament [15]. We observed that this nutritional strategy allowed higher stroke frequency during play, with decreased rates of perceived exertion. In this follow-up study we investigated the effects of this nutritional strategy on physical performance. Physical performance was assessed by a series of physical tests which determined strength, speed, power and endurance of the subjects following the end of the tennis tournament simulation in each condition (placebos and sports drinks). Our hypotheses were that physical performance would naturally decrease over the matches and that the sports drinks would limit this fatigue.

## Methods

### Trial design

This was a single-center, double-blind, placebo-controlled, cross-over trial conducted in France. It was performed according to Good Clinical Practice. This clinical trial was approved by the Southeast VI Ethics Committee for Human Research and by the French Health Products Safety Agency (2010-A00724-35). All procedures were in accordance with the ethical standards of the 1975 Helsinki Declaration, as revised in 1983. The study protocol was also registered at clinicaltrials.gov as NCT01353872.

### Subjects

Eight well-trained male tennis players volunteered to participate in this study (age 26.0 ± 5.7 years; height 1.84 ± 0.70 m; body mass 82 ± 11 kg). The major inclusion criteria were as follows: men aged 18 – 35 years with a body mass index ≥ 18.5 kg.m^−2^ and < 26 kg.m^−2^, nonsmoking or consuming less than 5 cigarettes per day, reporting a moderate caffeine intake (1–2 cups of coffee or equivalent per day), stable weight for at least one month before the beginning of the study, training at least twice a week, being involved in tennis-based training for at least three months prior to the beginning of the study, and figuring in the regional ranking tables drawn up by French Tennis Federation. Furthermore, participants also needed to have stable eating patterns during the month preceding the beginning of the protocol and had to agree to maintain these dietary habits throughout the study. Principal exclusion criteria included high-level sporting activities other than tennis and those necessary for their training, injury during the three months prior to the beginning of the study, diabetes, whether treated or detected at the inclusion visit, vegetarians and vegans, subjects with extreme eating habits and/or with a significant history of anorexia nervosa, bulimia or other eating disorders, a history of diseases that could disrupt our tests or subjects taking diuretics or other medications that we felt might interfere with our tests, and subjects consuming more than two alcoholic beverages per day (every day). Each candidate selected was fully informed of the purpose and risks associated with the procedures, and their written informed consent was obtained.

### Trial protocol

**Figure 1:**
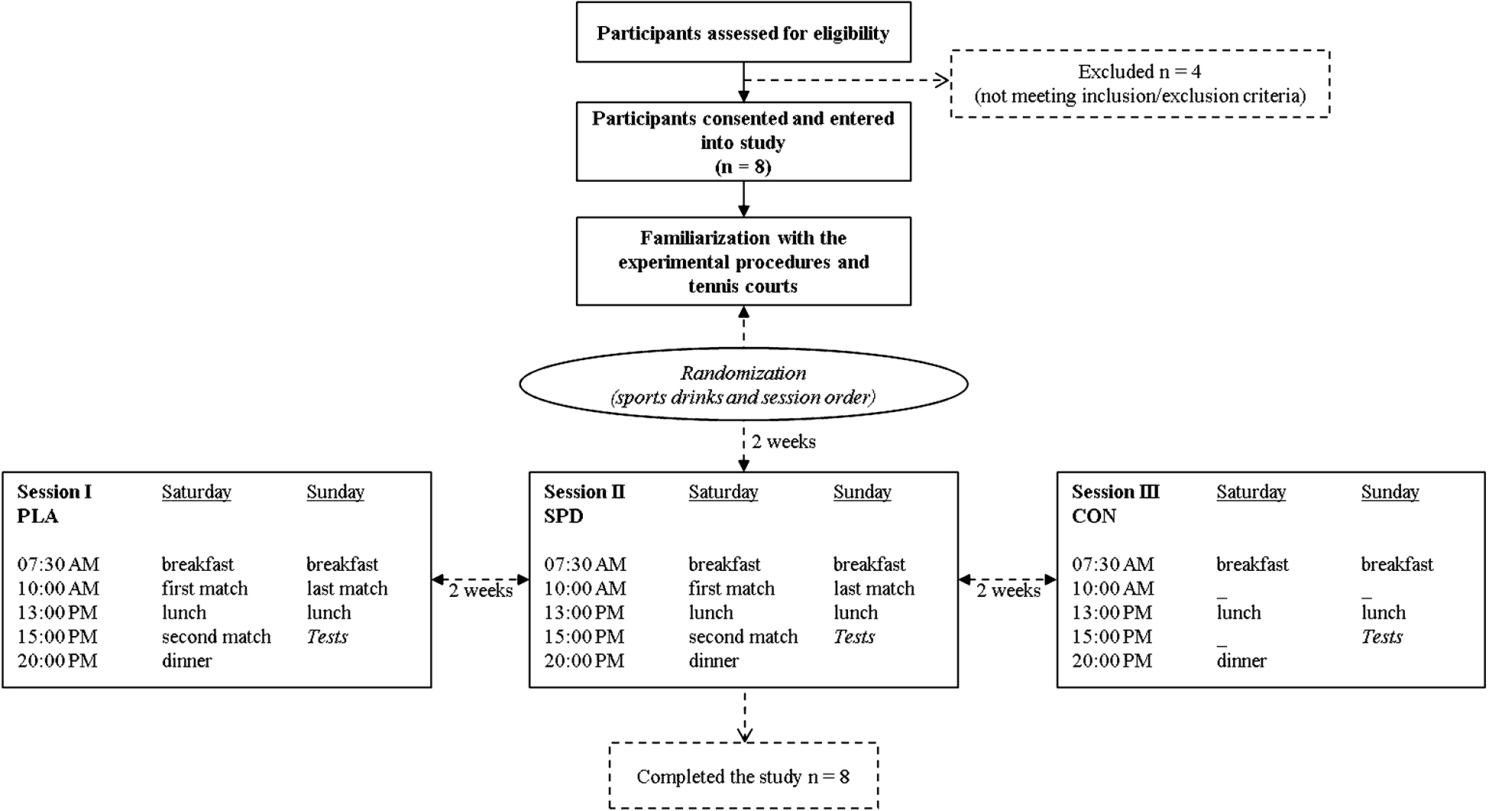
Experimental design and flow diagram of subjects’ passage through the study.

The experiment included three conditions, each of which consisted of a battery of physical performance tests: the first was after the “rest condition” (CON) and the other two were carried out following a tennis-tournament-type situation with matches played on 2 consecutive days during which the participants ingested either sports drinks (SPD) or placebos (PLA) (Figure 1). For each of the three conditions, the physical performance tests were performed at 3:00 PM on Sunday and 3 hours after the end of the last tennis match (for SPD and PLA). Each of the three test sessions was performed 2 hours and 30 minutes after a standardized meal. The order for the three conditions was randomized and each was separated by 2 weeks. All trials were performed on the same indoor, hard-surface (Greenset®) courts. The participants became familiar with the experimental procedures and courts during a training session which took place two weeks before their first test condition. The players were instructed to continue their usual dietary habits, refrain from any changes in food selections or exercise during the trial and asked not to consume any food supplements or functional foods during the study. From 48 hours before each session, training was not allowed and subjects were asked to refrain from consuming caffeine (coffee, tea, chocolate, cola), tobacco and alcohol. In order to minimize the influence of previous evaluation tests, the sequence of tests was selected to propose the most fatiguing tests at the end. The orders of testing and recovery times were the same in each condition: isometric handgrip strength, power (jump height), maximal 20-m sprints, repeated-sprint ability, maximal isometric strength and fatigability of knee and elbow extensors.

### Dietary protocol

To verify diet stability, the subjects were instructed to record their food intake during the 48 hours prior to each session. For this purpose, an instruction booklet containing daily menu examples was given to each subject who was trained by a dietician how to keep their intake diary during the inclusion meeting. Food diary records from each session were analyzed using Nutrilog® 2.10 software (Nutrilog®, Marans, Fance); their analysis revealed no significant difference in total caloric and macronutrient levels (data not shown). During each day of experiments, the diet was standardized as follows: fat 25%, protein 15% and carbohydrate 65%, plus 200 mL of mineral water at each meal. The total energy provided by breakfast, lunch and dinner was 3197, 4443 and 4841 kJ, respectively. This standardization of meals was done to minimize variations in pre-exercise glycogen stocks.

On match days three drinks were taken: before, during and after each match. The pre-match drink consisted of 500 mL of liquid (either a sports drink or a placebo) taken in the hour preceding the match. During the match subjects drank 750 mL/h of their test drink and 250 mL just after the match. The placebo drinks were specially developed to be similar in color and taste to the sports drinks. Moreover, subjects were informed that they were testing two sports drinks similar in appearance but different in composition and were therefore unaware that they may be taking a placebo. The drinks were kept at 8–10°C. Using a 10-point scale as a measure of gastrointestinal discomfort, the drinks were well tolerated (data not shown).

### Composition of drinks

The nutritional composition of the pre-match drink was as follows: fructose 36 g.L^−1^; maltodextrin 31 g.L^−1^; hydroxycitrate 300 mg.L^−1^; sodium citrate 7 g.L^−1^; caffeine 140 mg.L^−1^; vitamins B1 0.4 mg.L^−1^, B2 0.4 mg.L^−1^, B6 0.6 mg.L^−1^ (70 g.L^−1^, 1142 kJ.L^−1^). Composition of the placebo pre-match drink: citric acid, natural grapefruit flavor, sucralose, acesulfame potassium, sillicium dioxide, maltodextrin, beetroot juice concentrate (amount of traces), tartrazine (3.0 g.L^−1^, 35.8 kJ.L^−1^). Match drink: maltodextrin 31.6 g.L^−1^, dextrose 24.2 g.L^−1^, fructose 12.8 g.L^−1^, Branched-Chain Amino Acids 4 g.L^−1^, curcumin 250 mg.L^−1^, piperine 2.6 mg.L^−1^, caffeine 75 mg.L^−1^, sodium 884 mg.L^−1^, magnesium 100 mg.L^−1^, zinc 5 mg.L^−1^, vitamins C 15 mg.L^−1^, E 5 mg.L^−1^, B1 0.7 mg.L^−1^, B2 0.4 mg.L^−1^, B3 9 mg.L^−1^ (80 g.L_−1_, 1254 kJ.L^−1^). Placebo match drink: natural flavors, malic and citric acids, xanthan gum, acesulfame potassium, sucralose, silicium dioxide, yellow FCF, tartrazine (3.2 g.L^−1^, 50 kJ.L^−1^). Post-match drink: proteins 40 g.L^−1^; glucose 28.8 g.L^−1^; fructose 14.4 g.L^−1^; lipids 7.2 g.L^−1^; curcumin 680 mg.L-^1^; piperine 7.6 mg.L^−1^; sodium chloride 576 mg.L^−1^; potassium 156 mg.L^−1^; vitamins C 120 mg.L^−1^; E 20 mg.L^−1^; B1 5.6 mg.L^−1^; B2 3.3 mg.L^−1^; B3 72 mg.L^−1^; B6 4 mg.L^−1^ (64 g.L^−1^, 2144 kJ.L^−1^). Placebo post-match drink: natural flavors, titanium dioxide, tartrazine, maltodextrin, beetroot juice concentrate (amount of traces), citric acid, acesulfame potassium, sucralose, silicium dioxide (3.0 g.L^−1^, 68 kJ.L^−1^). All drinks were provided by Nutratletic (Aytré, France) and respected the current legislation for dietary products.

### Tennis tournament simulation

Each tournament took place over a weekend. On the experiment days (SPD and PLA sessions), the players arrived at the tennis club at 07 h00 after an overnight fast. A standardized breakfast was given to the subjects. The eight players were randomly divided into two groups of four players. Every player in each group played randomly against each of the three other players. Therefore, each player played three matches. The first game began at 10 h00 on Saturday, the second game at 15 h00 and the third match was scheduled at 10 h00 on Sunday. A standardized breakfast, lunch and dinner was given to the subjects at 07 h30, 13 h00 and 20 h00 respectively. To maintain the competitive aspect, but play for similar periods, the format of each game was adapted to last 2 h. In practice, the players played 3 sets using the No Ad scoring system to limit variability in the duration of the games. The first 2 sets were played in 6 games with a tiebreak in case of a tie at 6 all. At the end of the first two sets, the format of the third set was adjusted to obtain an estimated final match time of 2 h. If the duration of the first two sets was less than 1 h 20 min, the third set took place in 6 games, like the first 2. If the first 2 sets lasted between 1 h 20 min and 1 h 40 min, the third set was played in 4 games, with a tiebreaker played in the event of a tie at four games all. Finally, if the duration of the first 2 sets was above 1 h 40 min, the third set was replaced by a super tiebreak of 10 points. This protocol resulted in matches very close to 2 h in duration and with very low variability, while avoiding games played “in time”, which could have led to abnormal playing and have had a negative impact on the player motivation. No significant differences could be detected in the average duration of matches between the PLA and SPD sessions (data not shown).

### Isometric handgrip strength

Three consecutive measurements for isometric handgrip strength of the dominant hand were made with a calibrated dynamometer (TK200, Takei®, Niigata, Japan). The best performance was recorded for each subject. The apparatus was reset to zero before each measurement. The measurements were conducted under standardized conditions: subject seated, the shoulder adducted and neutrally rotated, with the forearm and wrist in a neutral position and the elbow at 90*°* flexion. The subjects were verbally encouraged to perform three, 3-s maximum voluntary contractions (MVC) separated by at least 3 min of recovery in between.

### Power (jump height)

All vertical jumps were performed using an optical measurement system (Optojump, Microgate®, Bolzano, Italy). A software program recorded jump height based on flight time. In order to ensure the validity of the test, participants were asked to have their knees as fully extended as possible and their ankles completely plantarflexed on both take-off and landing. Participants stood with their feet shoulder width apart and flat on the contact mat. The best jump from three attempts was recorded for both squat jumps (SJ) and countermovement jumps (CMJ). A 1-min recovery was provided between all jump trials. For both jump measurements, participants stood feet flat on the contact mat with hands on hips (no arm swing). For SJ measurements, participants held their knees flexed at 90° for two seconds, and were then told to jump as high as possible, avoiding the use of a stretch-shortening cycle as for CMJ. No specific instructions were given about the depth or speed of the countermovement for the CMJ test.

### Maximal 20-m sprints

The running speed of participants was evaluated with a 5- and 20-m sprint effort using photocells (Racetime2, Microgate®, Bolzano, Italy). The timing gates were positioned 5- and 20-m cross-wind from a pre-determined starting point. Participants were instructed to run as fast as possible along the 20-m distance from a standing start. Subjects started the test in their own time from a static position 30 cm behind the photocells, with timing starting once the beams of the first timing gate (0 m) were broken. The fastest time obtained from three trials was used in data analysis. There was a 2-min recovery period between trials. Time spent to cover 20-m was measured to the nearest 0.001 s.

### Repeated sprint ability

The repeated sprint ability test, which attempts to quantify fatigue by comparing actual performance to an imagined “ideal performance”, consisted of 6 times 24.69 m (3 times 8.23 m, corresponding to the width of the tennis court) of discontinuous sprints, interspersed with 30 s of walking recovery. The timing gates were positioned in the width of the court, at the opposite of the court’s two single lines. Subjects were instructed to run as fast as possible from one side to another 3 times from an initial standing start. Subjects started the test from a static position 30 cm behind the photocells, with timing starting once the beams of the first timing gate (0 m) were broken. Speed was measured to the nearest 0.001 s. A photoelectric cell timing system (Racetime2, Microgate®, Bolzano, Italy) linked to a digital chronoscope was used to record each sprint and rest interval time with an accuracy of 0.001 s. Fatigability (percent decrease in time between the fastest and slowest sprints) and sprint decrement score (Sdec) were calculated from sprint times using the following formula : Fatigue (%) = −((slowest sprint-fastest sprint)/fastest sprint)×100; Sdec (%) = −(((Sprint 1 time + Sprint 2 time + … + Sprint 6 time)/Best sprint time × number of sprints)-1)×100 [16].

### Knee and elbow extensors maximal isometric strength

The maximal isometric strength of the dominant knee extensors was measured from maximum voluntary contractions (MVC) performed on a custom-made ergometer. This ergometer was built in order to allow placement of the force transducer (Model F2712, 0- to 100-daN force range, Meiri Company, Bonneuil, France) at the level of the lateral malleolus and adjustment of the seat depth depending on the length of the thighs. The knee angle and the hip angle were set at 60° (0° is full extension). The knee was fixed at an angle of 60° of flexion since it has been demonstrated to be the angle of maximal isometric force generation for human muscles [17],[18]. The dominant leg was defined as the preferred kicking leg. Subjects were secured to the chair by a strap slung over the shoulders to avoid any compensatory movement of the trunk. Subjects were also instructed to grip the seat during the voluntary contractions to further stabilize the pelvis. The subjects were verbally encouraged to perform three, 3-s MVCs separated by at least 3 min of recovery. Isometric MVC strength was determined as the best of three reproducible measurements. During each trial, subjects were instructed to contract the muscle as strongly as possible. Isometric MVC torque of the knee extensor muscles (KE MVC torque) was calculated as the product of maximal force and moment leg length; the latter being measured from the lateral malleolus to the lateral femoral condyle in resting conditions using a tape measure.

The isometric MVC strength of the dominant (arm holding the racket) elbow extensors was measured on a custom-made, home-built ergometer. This ergometer was built in order to place the subjects in a seated position and pull a grip connected to a force transducer with the elbow flexed at 90°. During each test, subjects were instructed to keep their chest in an upright position to avoid any compensatory movement of the trunk. The experimental protocol was the same as that previously described for the knee extensors. The subjects were encouraged to perform three, 3-s isometric MVCs separated by 3-min resting periods. Isometric MVC strength was determined as the best trial from three reproducible measurements. Isometric MVC torque of elbow extensors (EE MVC torque) was calculated as the product of maximal force and moment arm length, the latter being measured from the lateral epicondyle of the elbow to the ulna head in resting conditions using a tape measure. Force output was measured using a calibrated force transducer (Model F2712, 0- to 100-daN force range, Meiri Company, Bonneuil, France) and transmitted to a PC using an analog/digital card (National Instruments, NI USB-6211, France).

### Knee and elbow extensors fatigability

The subjects performed a 90-s sustained isometric contraction at 25% MVC in order to evaluate the muscle fatigability of the main knee and elbow extensor muscles. Visual feedback about force was provided to the subject during the sustained contraction. Electromyographic signals (EMG) of the superficial heads of the knee extensors (*vastus lateralis*, *vastus medialis* and *rectus femoris*) and the *triceps brachii* muscle (medial and lateral heads) were recorded throughout the 90-s sustained contraction. EMG was quantified in the time domain using the Root Mean Square value (RMS). All the RMS values recorded during the 90-s contraction were normalized to a percentage of maximal Root Mean Square value (RMS_max_) of the best MVC trial for each muscle.

### Electromyography

The EMG signals were recorded using bipolar silver chloride surface electrodes (Kendall, Arbo, Tyco Healthcare, Neustadt, Germany) during the MVCs and the fatigability test. The recording electrodes were taped lengthwise on the skin over the muscle belly following SENIAM recommendations [19], with an inter-electrode distance of 20 mm. The position of the electrodes was marked on the skin so that they could be fixed in the same place during the following test session. The reference electrode was attached to the patella or to the elbow. Low impedance (Z < 5 kΩ) at the skin-electrode surface was obtained by shaving, abrading the skin with thin sand paper and cleaning with alcohol. Electromyographic signals were amplified with a bandwidth frequency ranging from 10 Hz to 500 Hz and simultaneously digitized together with force signals using an acquisition card (National Instruments, NI USB-6211, Nanterre, France) and a custom made software (MatLab Version 7.5.0, R2007b). The sampling frequency was 1000 Hz.

### Statistical analyses

Data are reported as mean values ± standard deviation (SD). The statistical analyses were done using GraphPad PRISM® 5.01 software (La Jolla, USA). A p-value < 0.05 was considered significant. Two-way ANOVA were used when the interaction between time and condition effects was tested (EMG data). Other endpoints were analyzed using non-parametric tests. To test for the condition effect (CON, PLA, SPD), the Kruskal-Wallis one-way test was used. In case of significant difference, the Wilcoxon signed-rank test was performed to compare all pairs of conditions.

## Results

Eight subjects completed all three different test conditions without experiencing any complications. During the three test sessions, environmental conditions were not significantly different: ambient temperature was: 27.1 ± 0.4, 27.5 ± 0.5 and 28.0 ± 0.4°C in the CON, PLA and SPD sessions, respectively. The relative humidity was 38.0 ± 2.7, 40.0 ± 3.0 and 41.0 ± 3.3% in the CON, PLA and SPD trials, respectively.

### Isometric handgrip strength

**Figure 2:**
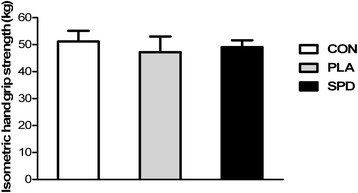
**Mean (±SD) isometric hand grip strength with the dominant hand in the 3 conditions (CON, PLA and SPD).** Inter-group analysis was carried out using the Kruskal-Wallis one-way analysis; no statistical difference was found.

Average handgrip strength values for the CON, PLA and SPD were 51.18 ± 1.36, 47.23 ± 2.01 and 49.08 ± 0.88 kg respectively, with no significant difference between the 3 conditions (Figure 2).

### Power (jump height)

**Figure 3:**
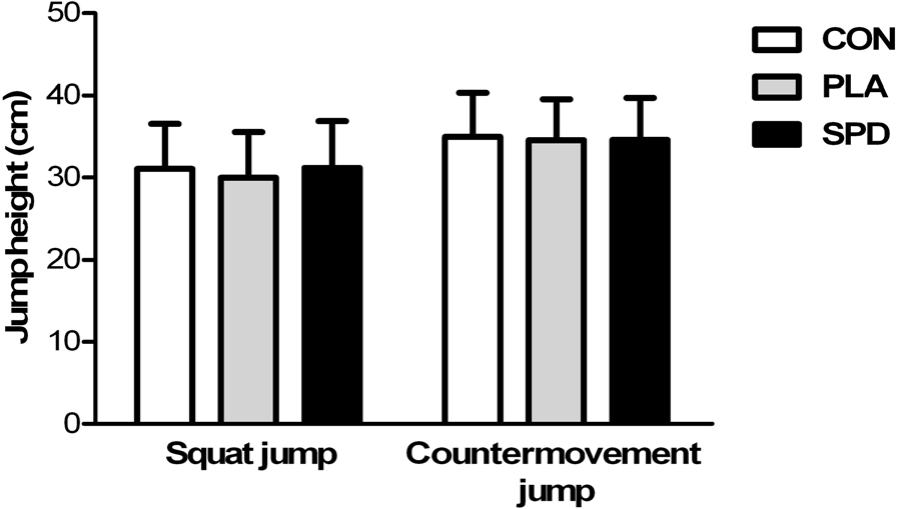
**Mean (±SD) jump height for the squat (SJ) and countermovement (CMJ) jumps in the 3 conditions (CON, PLA and SPD).** For SJ and CMJ, inter-group analysis was carried out using the Kruskal-Wallis one-way analysis; no statistical differences were found.

Average CMJ height values for the CON, PLA and SPD were 34.98 ± 1.87, 34.55 ± 1.75 and 34.60 ± 1.78 cm, respectively, with no significant differences between these 3 conditions (Figure 3). Average SJ height values for the CON, PLA and SPD were 31.05 ± 1.91, 29.98 ± 1.93 and 31.20 ± 1.97 cm, respectively, with no significant difference between the three conditions (Figure 3).

### Maximal 20-m Sprints

**Figure 4:**
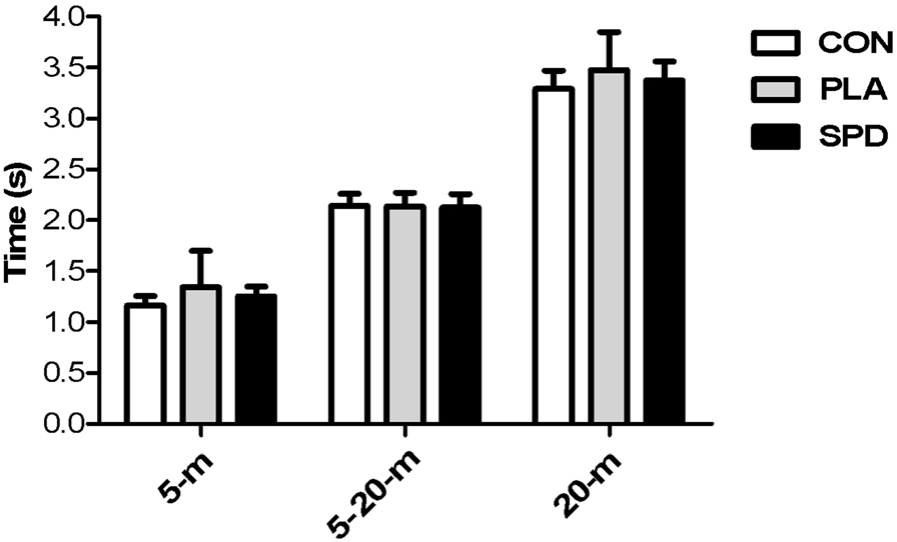
**Average (±SD) time spent to cover 5-, 5 to 20- and 20-m sprint values for the 3 conditions (CON, PLA and SPD).** Inter-group analysis was carried out using the Kruskal-Wallis one-way analysis; no statistical difference was found.

Average 5-m sprint time values for the CON, PLA and SPD were 1.16 ± 0.03, 1.34 ± 0.12 and 1.26 ± 0.03 s, respectively. Average 5 to 20-m sprint time values for the CON, PLA and SPD were 2.14 ± 0.04, 2.14 ± 0.05 and 2.13 ± 0.05 s, respectively. Average 20-m sprint time values for the CON, PLA and SPD were 3.29 ± 0.06, 3.48 ± 0.13 and 3.37 ± 0.06 s, respectively. No significant differences between the three conditions were observed for any of the sprint times (Figure 4).

### Repeated sprint ability

**Figure 5:**
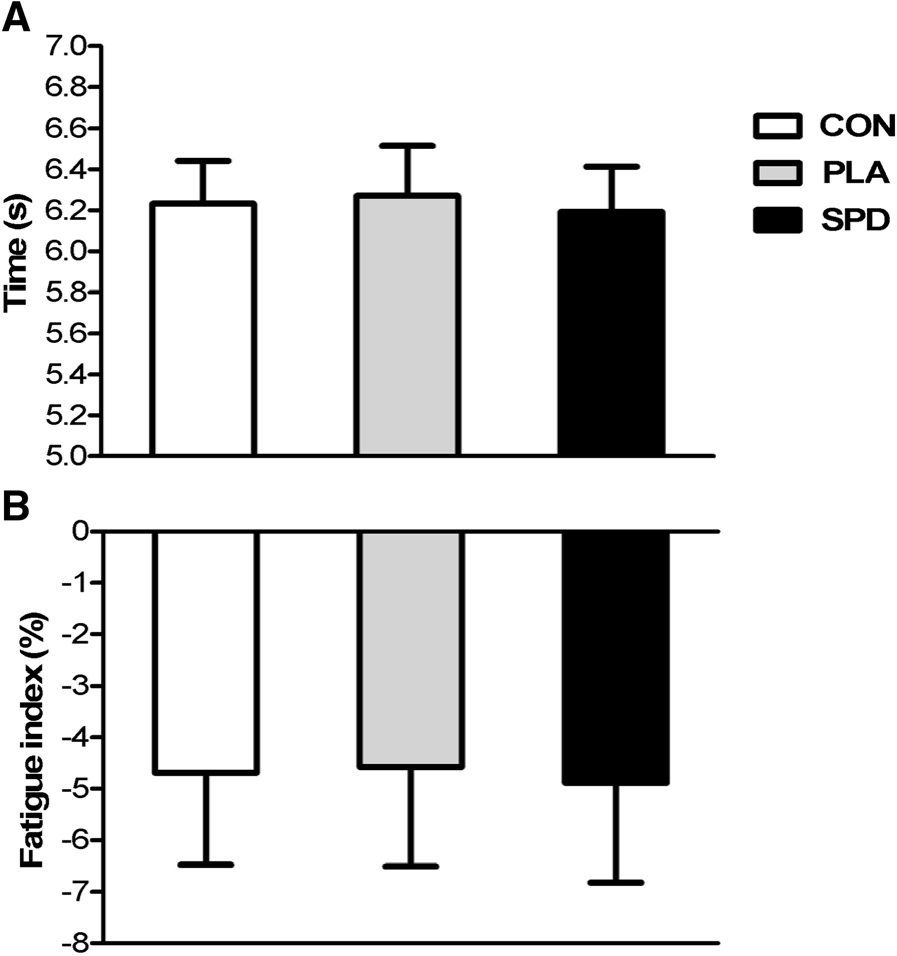
**Repeated sprint best time and fatigue index.** Average (±SD) best performance for the 3 x 8.23 m repeated sprints in the 3 conditions (CON, PLA and SPD) (Panel **A**). Mean (±SD) fatigue index in the 3 conditions (Panel **B**). Fatigue index was calculated by the percent decrease in time between the fastest and the slowest sprints. Inter-group analysis was carried out using the Kruskal-Wallis one-way analysis; no statistically difference was found.

Average best performance for the 3 × 8.23 m (24.69 m) repeated sprint test for the CON, PLA and SPD were 6.23 ± 0.21, 6.27 ± 0.24 and 6.19 ± 0.22 s, respectively, with no significant difference between the three conditions (Figure 5, Panel A). The fatigue index was also not significantly different between conditions (−4.69 ± 0.63, −4.58 ± 0.68 and −4.88 ± 0.68% for CON, PLA and SPD respectively) (Figure 5, Panel B). The sprint decrement score was also not different between the three conditions (−3.21 ± 0.50, −2.70 ± 0.58 and −2.98 ± 0.41% for CON, PLA and SPD respectively).

### Knee and elbow extensors maximal isometric strength

**Figure 6:**
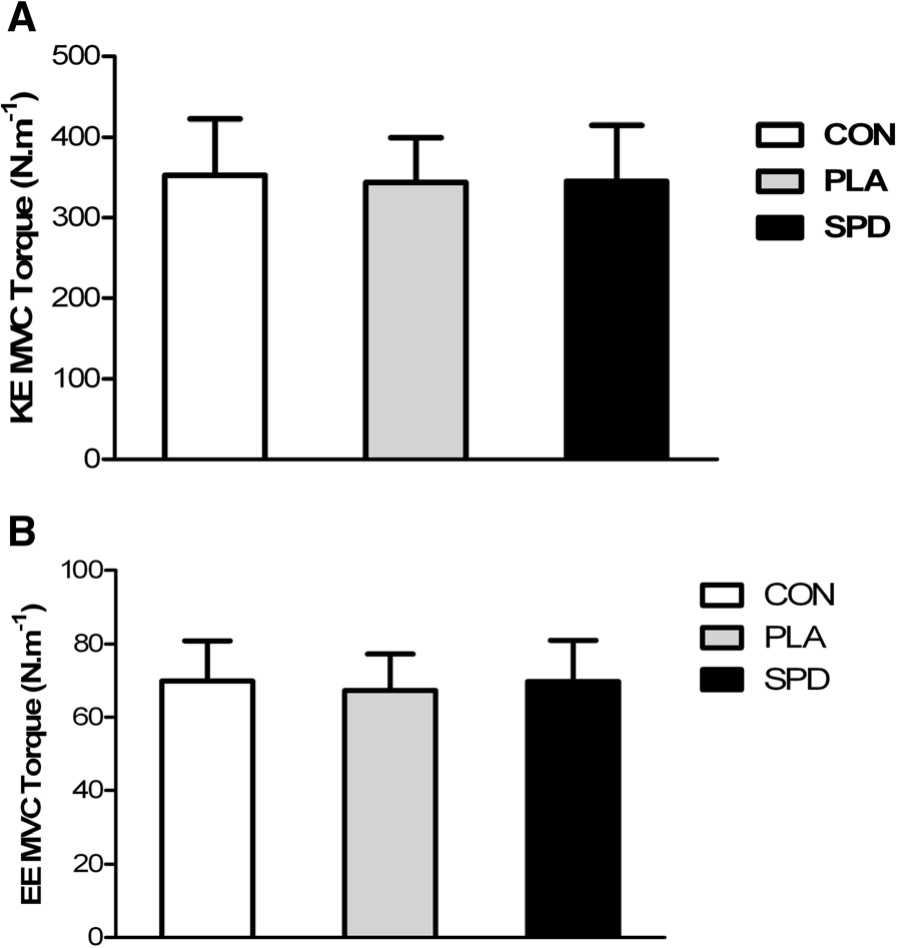
**Mean (±SD) isometric maximal voluntary contraction torque of knee extensors (KE MVC Torque, Panel A) and of elbow extensors (EE MVC Torque, Panel B) in the 3 conditions (CON, PLA and SPD).** For KE MVC Torque and EE MVC Torque, inter-group analysis was carried out using the Kruskal-Wallis one-way analysis; no statistical differences were found.

Analysis of variance revealed no significant difference in knee extension and elbow extension MVC torque between the three experimental conditions (Figure 6).

### Knee and elbow extensors fatigability

**Figure 7:**
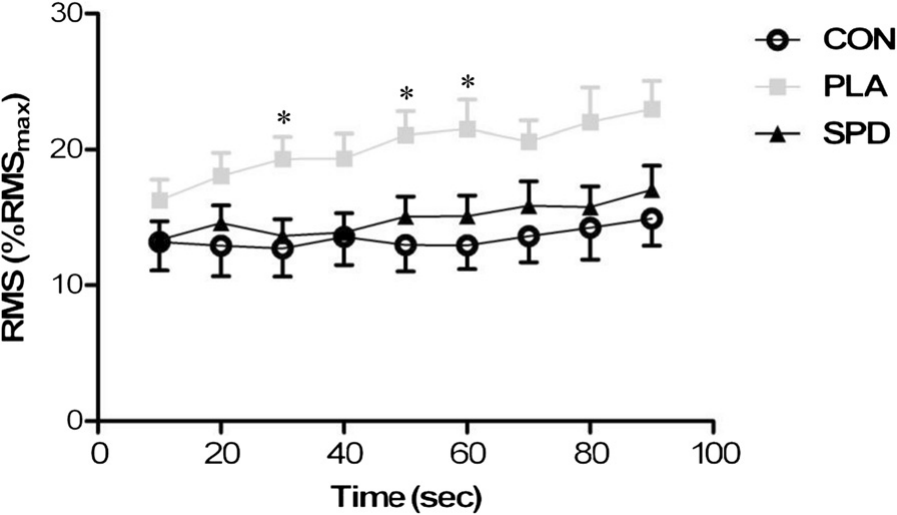
**Time course of root mean square (RMS) values (expressed as a percentage of the maximal RMS of the best MVC trial) of the*triceps brachii*muscle (lateral head) throughout the 90-sec time trial.** Results are expressed as mean values ± SD in the three conditions (CON, PLA and SPD). Two-way ANOVA was used (time and condition); time effect: p < 0.001, condition x time effect: p = 0.0167. Bonferroni post hoc analysis showed significant difference between PLA and SPD for time 30-, 50- and 60-sec (p < 0.05). Tendencies were observed for time 40-sec (p = 0.07), 80-sec (p = 0.08) and 90-sec (p = 0.07).

Electromyographic changes throughout the 90-s sustained isometric contraction were similar in the 3 conditions for all muscles, except for the lateral head of the *triceps brachii* whereby RMS values increased significantly less in the SPD group compared to the PLA group (Figure 7). No significant difference was observed for the *triceps brachii* between the SPD and CON conditions.

## Discussion

The objective of this study was to evaluate the effects of a nutritional strategy on the physical performance of competitive tennis players. This strategy consisted of taking a pre-match drink, a match-drink and a post-match drink during every match of a simulated tennis tournament. Based on data in the literature, showing that a prolonged tennis match could induce muscle fatigue [20],[21], our first hypothesis was that repeated tennis matches would induce a decrease in physical performance even after a few hours of recovery compared to the resting condition. Since some studies have also demonstrated that carbohydrate supplements during prolonged tennis matches delays the onset of fatigue [4],[5],[8]–[10], our second hypothesis was that drinking sports beverages before, during and after each tennis match would limit the decrease in physical performance compared to conditions where the only fluid intake was water. The main results show that playing three simulated tennis matches in a thirty-six-hour period did not significantly decrease any of the physical performance measures 3 h after the last match.

Various studies have shown that prolonged tennis playing in competitions leads to the development of muscle fatigue that may impair skilled performance on the court [3]–[6]. However, all of these studies conducted performance tests during or immediately after the match. Given the characteristics of tennis tournaments, *i.e.* several matches in a limited time-frame interspersed with short recovery periods, it is important to consider whether these consecutive matches would finally result in decreased physical performance and whether ingesting sports drinks before, during and after each match would limit fatigue, facilitate recovery and so favor improved performance in subsequent matches. Considering that nutritional strategies can have an important influence on the capacity to recover [14],[22], notably influencing muscle and hepatic glycogen stores [23], we have been careful in this study to precisely control the amount and type of nutrients ingested during the meals taken by the players in the different conditions studied. Thus the breakfasts, lunches and dinners eaten on study days were standardized and identical for each of the conditions.

The results of our study show that after playing three 2-hour matches within thirty-six hours, only 3 hours of passive recovery (including the ingestion of a standardized lunch) was sufficient to observe no significant decrease in physical performance parameters, compared to the rest condition. The only significant difference in physical performance was the increase in RMS values during the 90-s sustained isometric contraction at 25% MVC for the lateral head of the *triceps brachii* in the PLA condition compared to the CON condition. The increased RMS value under the placebo condition is an index of fatigue and indicative of the greater number of motor units required to maintain an equivalent level of force [24].

This study, the first to assess the influence of repeated tennis matches on physical performance, suggests that when the length of a match does not exceed 2 hours, when balanced meals are taken between matches, and when hydration during matches is sufficient, there is no major deleterious impact on physical performance of the lower-limb muscles. It has already been suggested that skilled tennis performance, which can be affected by prolonged match-induced fatigue [3],[6], quickly returns to normal [21],[25]. We can hypothesize that, if the measurements of physical performance had been carried out immediately after the end of the last match of a tournament, a significant decline in performance parameters would have been observed. For example, two recent studies [26],[27] showed a decrease of 9 - 15% in the plantar flexor muscles’ MVC immediately after 3-hour tennis matches. Nevertheless, two-hour tennis matches are not always associated with decreased performance. Indeed, McRae et al. were not able to show any significant decrease in a specific tennis skill-test following a 2-hour tennis match [10]. We can hypothesize that a succession of longer matches and/or more intense and/or performed under more constraining environmental conditions would have induced a decrease in physical performance even after several hours of recovery, but more studies are needed to address this hypothesis. Moreover, most of the studies exploring muscle fatigue following tennis matches have used an isometric device [26]–[32]. To date, only one study has evaluated the impact of tennis practice on muscle performance using isokinetic dynamometer in elite young tennis players [33]. They found that a 90 min practice session induced a 9 to 13% decrease in the knee extensors and flexors of the contractile joint moments evaluated at 60 and 180°.s^−1^. Therefore, it would be particularly interesting to conduct more studies evaluating fatigue following tennis matches and practice sessions using isokinetic measurements, which represent more closely tennis activity muscle contraction pattern.

In this study, we evaluated physical performance through some simple tests of speed, strength, power and endurance. However, it is conceivable that complementary tests might have revealed fatigue, or that a specific assessment of tennis performance would have demonstrated a drop in performance. One explanation for the fact that the only fatigue observed in our study concerned the *triceps brachii* muscle could be the fiber composition of this muscle, as it has been recognized that this influences muscle fatigue [34]. It has also been shown that the *triceps brachii* muscle has a fast profile, with less than 20% of type I fibers [35], while the quadriceps muscle has a more mixed profile with more than 50% of type I fibers [36]–[38].

The second objective of this study was to assess whether supplementation with sports drinks before, during and after each match, compared with water alone, could limit the negative impact of the succession of matches on physical performance of the players. Since consecutive matches induced little or no drop in performance during the tests performed three hours after the last match, it is not surprising to observe almost no difference between the placebo and drinks conditions. Interestingly, in our study the only fatigue observed in the placebo condition compared with the rest condition (an increase in RMS of the *triceps brachii* muscle), was counteracted when the players were supplemented with sports drinks. The main active ingredients of the drinks consumed by the players were carbohydrates (pre-match drink, match-drink and post-match drink), caffeine (pre-match drink and match-drink), and proteins (match-drink and post-match drink). Some studies have already demonstrated the potential of carbohydrates and caffeine supplementation to positively affect performance of tennis players [4],[5],[8]–[10], while proteins have only been suggested [21]. In the context of repeated matches with short recovery periods, it is at least conceivable that a decrease in glycogen stocks may contribute to the development of muscle fatigue, and that supplementation with carbohydrate before, during and after each match could promote the use of exogenous substrates and the rate of resynthesis of glycogen stocks between matches and therefore finally enable better maintenance of performance over repeated matches. Given that a drop in tennis performance has been observed during extended matches (>3 h), further research is needed to investigate whether the current nutritional supplementation strategy would more effective under such conditions.

In conclusion, this study demonstrates that playing three 2-hour tennis matches in a day and a half does not induce any significant decrease in physical performance of the lower-limb muscles three hours after the end of the last match, when water-based hydration is sufficient and the meals are well-balanced. The only fatigue observed in the placebo condition compared with the rest condition involved the *triceps brachii* muscle, and this fatigue was counteracted when the players were supplemented with sports drinks, which allows one to hypothesize that this type of nutritional strategy could be effective in the more extreme conditions that occur during competitive tennis tournaments. Further studies are needed to address this hypothesis which could lead to interesting practical recommendations for players and coaches.

## Competing interests

Nicolas Aubineau and Sébastien L Peltier are employees of Laboratoire Lescuyer-Nutratletic. Jean-François Lescuyer is the general director of the company.

This trial was carried out by Laboratoire des Adaptations Métaboliques à l’Exercice en conditions Physiologiques et Pathologiques (AME2P) and Laboratoire Lescuyer-Nutratletic as a joint venture.

The other authors have no competing interests.

## Authors’ contributions

TB: conception and design of the study, acquisition of data, analysis and interpretation of data, drafting manuscript. SR: conception and design of the study, acquisition of data (electromyographic measures), analysis and interpretation of data (electromyographic measures), drafting manuscript. PL: conception and design of the study, acquisition of data, analysis and interpretation of data, revising manuscript. LM: acquisition of data, dietary protocol management, revising manuscript. GE: acquisition of data, analysis and interpretation of data, revising manuscript. ED: conception and design of the study, acquisition of data, revising manuscript. VM: analysis and interpretation of data (electromyographic measures), revising manuscript. DB: design of the study, revising manuscript. NA: analysis and interpretation of data, revising manuscript. JL: conception and design of the study, revising manuscript. MD: conception and design of the study (main clinical investigator), acquisition of data, revising manuscript. PS: conception and design of the study (main project coordinator), acquisition of data, analysis and interpretation of data, drafting manuscript. SP: conception and design of the study (main project coordinator), analysis and interpretation of data, statistical analysis, drafting manuscript. All authors have read and approved the final manuscript.
